# Simultaneous Resection of Pituitary Adenoma and Contiguous Cavernous Sinus Meningioma: A Case Report

**DOI:** 10.7759/cureus.49106

**Published:** 2023-11-20

**Authors:** Fernando S Geldres, Luis Flores, Relix Huaman, Andy Zare, Yoel Quispe

**Affiliations:** 1 Department of Neurological Surgery, Victor Lazarte Echegaray Hospital, Trujillo, PER

**Keywords:** tumor, transcranial, meningioma, neoplasm, pituitary

## Abstract

Cavernous sinus meningiomas and pituitary adenomas, among the most prevalent benign intracranial neoplasms, rarely occur simultaneously. The proximity of these tumors in a shared location adds to the rarity of diagnosis, posing a unique challenge in their management. This report describes the case of a 52-year-old woman who presented with seizures, severe headaches, diplopia, and rapidly worsening vision. Imaging uncovered two distinct lesions: a pituitary adenoma located in the sellar region and an adjoining cavernous sinus meningioma. The patient underwent a single-stage transcranial approach, enabling the complete resection of both tumors. Histopathological analysis subsequently affirmed the initial diagnosis. Following the surgery, the patient experienced an enhancement in visual acuity without any postoperative complications. We emphasize the significance of a meticulous review of preoperative images, a crucial step that informs our tailored approach for each patient. At times, unique associations come to light. In this particular case, we proudly present the adept resection of adjacent tumors using transcranial surgery, underscoring a favorable recovery marked by the absence of post-procedural neurological deficits.

## Introduction

Meningiomas and pituitary adenomas stand out as among the most prevalent benign intracranial neoplasms [1,2]. However, their simultaneous occurrence is an uncommon phenomenon. While it is more frequently observed that pituitary adenomas extend into the cavernous sinus, the coexistence of distinct contiguous tumors remains a rarity. The similarity in the clinical manifestations of both tumors compounds the challenge of accurate radiologic diagnosis and effective management [3]. In this context, we present a compelling case involving the simultaneous presence of a pituitary adenoma and a meningioma within the cavernous sinus. Notably, these tumors were successfully and entirely excised through a singular transcranial approach in a single surgical stage.

## Case presentation

History and examination

A 52-year-old female patient presented with a complex array of symptoms that unfolded over a two-week duration, warranting immediate medical attention. Notably, she experienced recurrent seizures, characterized by focal motor manifestations, and reported an intense, throbbing severe headache that persisted despite standard analgesic measures. Additionally, the onset of diplopia was observed, further adding to the multifaceted nature of her symptoms. Intriguingly, the patient reported a rapid and alarming deterioration in her vision, prompting concern about potential underlying neurological or ophthalmic pathologies. Her medical history provided insight into a background of controlled hypertension managed with oral antihypertensives. Upon conducting a meticulous physical examination, a series of pertinent findings emerged. Bilateral temporal hemianopia was confirmed through perimetry testing, signifying a specific visual field defect that raised questions about the localization of the pathology. Furthermore, the assessment of visual acuity in the left eye revealed a noteworthy diminution, measuring at 20/200. 

The remainder of the neurological and endocrinological assessments yielded normal results, including the following serum levels: prolactin (PRL) 15 ng/ml, growth hormone (GH) 0.15 ng/ml, insulin-like growth factor I (IGF-I) 120 ng/ml, adrenocorticotropic hormone (ACTH) 22 pg/ml, cortisol 21.6 g/ml, thyroid-stimulating hormone (TSH) 1.32 UI/ml, thyroxine (T4) 1.88 ng/dl, follicle-stimulating hormone (FSH) 3.8 mUI/ml, and luteinizing hormone (LH) 2.7 mUI/ml.

Imaging studies

**Figure 1:**
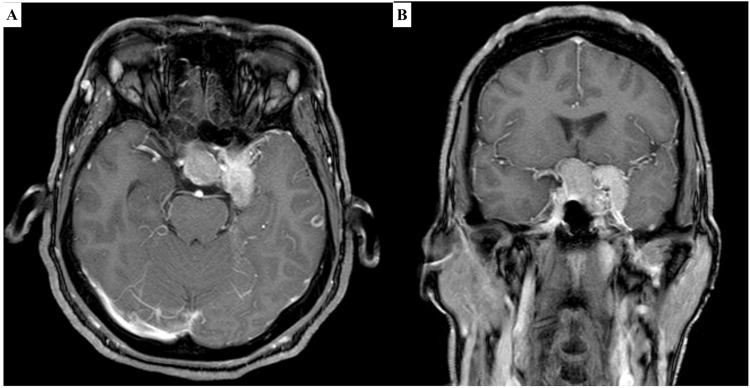
Preoperative magnetic resonance imaging The contrast-enhanced sagittal T1-weighted image (A) and coronal T1-weighted image (B) vividly depict an intrasellar mass, measuring approximately 19x19.8x21 mm with a subtle suprasellar bulge and homogeneous enhancement, suggestive of a Knosp 2 pituitary adenoma. Additionally, an adjacent left paresellar cavernous sinus-involving lesion is evident, measuring 16.5x23.5x26 mm. This lesion showcases a heightened contrast enhancement, prominently encasing the left internal carotid artery. The distinct characteristics observed raise the suspicion of a meningioma.

A non-contrast head CT revealed a sellar mass extending into the suprasellar and left parasellar region. CT scans of the thorax, abdomen, and pelvis were negative for any masses. A gadolinium-contrast-enhanced MRI (Figure 1) revealed a notable intrasellar mass extending into the suprasellar region, consistent with the characteristics of a pituitary adenoma. However, a distinctive observation emerged as a contiguous left lesion within the left cavernous sinus displayed a more pronounced and intense contrast enhancement. This heightened contrast enhancement raised a diagnostic red flag, prompting a closer examination. The unique contrast enhancement patterns between the intrasellar mass and the left cavernous sinus lesion triggered concerns about the potential coexistence of two distinct lesions. The preliminary suspicion centered around the possibility of encountering not only a non-functional pituitary adenoma but also a left cavernous sinus meningioma, emphasizing the need for thorough evaluation and precise characterization of both lesions for an accurate diagnosis and tailored treatment approach.

Operation

The patient chose surgery, specifically a transcranial approach, which was agreed upon. Utilizing a left transzygomatic approach with extradural clinoidectomy, we gained access to both the left cavernous sinus lesion and the sellar mass. Upon opening the Sylvian fissure, we encountered a highly vascular fibrous tumor originating from the lateral wall of the cavernous sinus. This tumor exhibited significant involvement with the left internal carotid artery and the left optic nerve. Following a meticulous microdissection, the tumor was delicately separated from the internal carotid artery and optic nerve. The outcome was a successful gross total resection, offering effective decompression for the optic nerve while ensuring the preservation of the structural integrity of the carotid artery vasculature. This phase concluded with precise bipolar coagulation of the cavernous sinus's lateral wall.

In the subsequent phase of the operation, our attention was directed towards the intricate sellar-suprasellar mass. The meticulous opening of the sellar diaphragm revealed a soft tumor with characteristic features indicative of a pituitary adenoma. Employing precision techniques, the tumor was promptly and meticulously extracted using a suction device and pituitary curettes. Both surgical specimens were diligently collected and sent for pathological examination. 

Pathological findings

**Figure 2:**
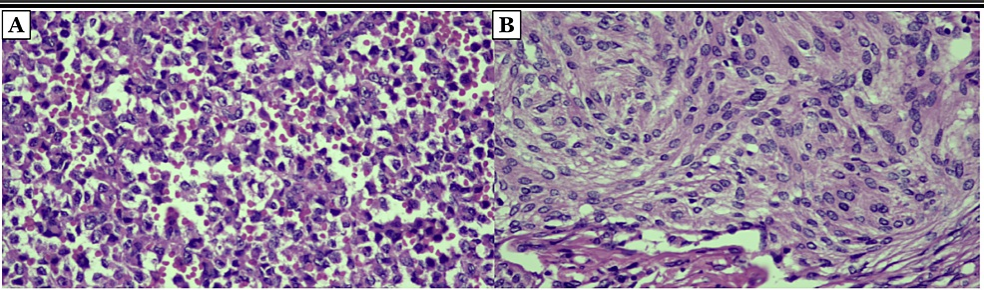
Postoperative histopathological examination Reveals a dual pathology, comprising a meningioma and a non-functioning pituitary adenoma. (A) The pituitary adenoma manifests as a cluster of irregular nuclei, displaying pleomorphic and eosinophilic cytoplasmic features indicative of its non-functioning nature. Notably, these cells exhibit positivity for neuron-specific enolase, as evidenced in the 400x photomicrograph; (B) Concurrently, the meningioma component is characterized by oval, fusiform, and lobulated cells with oval nuclei and thin chromatin, as revealed in the 400x photomicrograph. Further immunohistochemical profiling indicates progesterone receptor positivity, classifying it as a transitional meningioma of WHO grade I.

The histopathological features of the sellar and parasellar lesions were thoroughly examined (Figure 2), leading to the conclusive diagnosis of a pituitary adenoma for the sellar region. Additionally, the left parasellar lesion involving the cavernous sinus was identified as a transitional meningioma, classified as WHO grade I.

Postoperative course

**Figure 3:**
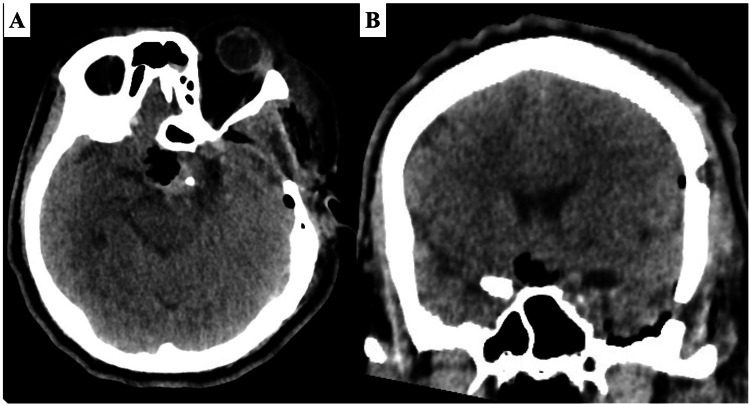
Post-operative non-contrast CT Axial (A) and coronal (B) images show a reassuring absence of any residual tumor.

Within 24 hours postoperative, non-contrast CT imaging (Figure 3) revealed a successful gross total resection. The patient exhibited enhanced visual acuity, and pituitary function remained consistent with preoperative levels. Throughout the follow-up period, there was partial improvement in the bitemporal visual field defect. Remarkably, no postoperative complications were observed.

## Discussion

Meningiomas stand as the predominant benign intracranial tumors in adults, constituting 37% of all primary central nervous system tumors. Among them, 15% originate in the parasellar region, with a mere 1% located in the cavernous sinus [4,5]. Concurrently, pituitary adenomas, representing 10-25% of intracranial tumors, exhibit parasellar extension into the cavernous sinus in around a quarter of surgical cases [6,2]. The coexistence of these distinct intracranial tumors is exceedingly rare, with a limited number of documented instances delineating the association between meningiomas and pituitary adenomas [3].

This correlation has been the subject of prior investigations, revealing a pivotal physiopathological mechanism contributing to the development of a meningioma subsequent to radiotherapy for pituitary adenoma [7]. Notably, in such instances, meningiomas often manifest within the irradiated field and are particularly linked to GH-secreting adenomas, hinting at a potential role of GH in inducing meningioma growth [8]. However, this mechanistic explanation falls short of elucidating the association with non-functioning adenomas, as observed in the case we present. The possibility of this connection being a coincidence prompts consideration for further comprehensive studies to explore the true nature of this association [9].

The encounter with a rare presentation of collision tumors introduces challenges in preoperative clinical and imaging diagnosis. This complexity may occasionally result in confusion, as the two tumors can mimic a singular lesion. Furthermore, in specific instances, variations in MRI signals may erroneously suggest the presence of distinct tumors due to differences in signal intensities. Emphasizing the importance of meticulous preoperative clinical and imaging assessments becomes imperative in navigating these challenges, facilitating a comprehensive understanding of the pathology and contributing significantly to an accurate preoperative diagnosis [10]. However, histological results invariably emerge as the linchpin for a definitive diagnosis, as preoperative findings often lack the precision required for accurate characterization [11]. In the case under consideration, the preoperative MRI evoked suspicions regarding the concurrent presence of a cavernous sinus meningioma and pituitary adenoma, as revealed by distinct contrast enhancement intensities between the two lesions. Subsequent surgical exploration served to substantiate these suspicions, with microscopic observations identifying discernible differences in tumor color and consistency between the sellar and parasellar lesions. The convergence of this clinical evidence compellingly suggested the coexistence of two distinct tumors, a hypothesis subsequently validated by the pathological results.

The optimal approach for managing this tumor presentation remains a subject of debate. In a recent literature review by Bao et al., encompassing approximately 20 cases featuring both pituitary adenomas and parasellar/suprasellar meningiomas, it was found that diverse strategies were employed, including the transcranial approach, endoscopic endonasal approach, and combinations of these methods [3]. Interestingly, the transcranial approach appeared to yield a higher rate of gross total resection. Typically, these tumors are managed independently, with the pituitary adenoma often addressed first, predominantly through a transsphenoidal approach. Subsequently, the meningioma is treated separately, commonly via a transcranial approach.

The consensus statement ratified by the European Association of Neurosurgical Societies Skull Base Section advocates for the transcranial approach in cases where surgery is deemed necessary due to compression of the optic nerve, brainstem, or temporal lobe [4,8]. Importantly, these clinical indications closely aligned with the presentation observed in our patient, thereby influencing our decision to employ the transcranial approach.

To the best of our knowledge, the extant literature encompasses only a solitary documented case elucidating the simultaneous presence of a pituitary adenoma and a cavernous sinus meningioma. The successful management of this case employed an endoscopic endonasal approach, culminating in a favorable neurological outcome [3]. In juxtaposition, the current case differed in its approach, involving a single-stage transcranial surgical technique. This method facilitated a gross total resection, resulting in a successful recovery devoid of neurological complications. This comparative analysis highlights the distinct surgical strategies employed in addressing such complex coexisting pathologies.

Acknowledging the inherent challenges posed by cavernous sinus meningiomas, these lesions are frequently characterized by a broad dural attachment and the potential encasement of nearby vessels and cranial nerves. In light of the heightened surgical complexities and risks involved, we assert that a judicious approach involves considering partial or subtotal resection, followed by the implementation of radiosurgery. This strategic course of action is deemed a safer and prudent choice in numerous instances, aiming to optimize patient outcomes while mitigating the potential complications associated with more extensive surgical interventions.

## Conclusions

In this report, the exceptional rarity of concurrent pituitary adenomas and meningiomas was explored, particularly within the cavernous sinus, a seldom observed phenomenon. The proximity of these tumors, coupled with their analogous MRI characteristics, intensifies the complexity of the diagnostic challenge. The optimal surgical approach remains a debated topic, and our case contributes to this ongoing discourse. Amidst varied strategies reported in the literature, the transcranial approach stands out as a favorable option, especially when guided by clinical indications such as optic nerve, brainstem, or temporal lobe compression. The successful transcranial resection of both tumors not only highlights the feasibility of this surgical approach but also results in favorable neurological outcomes.

Despite its rarity, our case augments the growing body of knowledge in the field, providing insights into both diagnostic and therapeutic aspects of these intricate intracranial presentations. Continued research and comprehensive studies are imperative to refine our understanding of the association between meningiomas and pituitary adenomas and to establish standardized approaches for their management.
